# Factors associated with the utilization of diagnostic tools among countries with different income levels during the COVID-19 pandemic

**DOI:** 10.1186/s41256-023-00330-1

**Published:** 2023-10-27

**Authors:** Shuduo Zhou, Xiangning Feng, Yunxuan Hu, Jian Yang, Ying Chen, Jon Bastow, Zhi-Jie Zheng, Ming Xu

**Affiliations:** 1 Department of Global Health, School of Public Health, Peking University, 38 Xue Yuan Road, Haidian District, Beijing, 100191 China; 2 Institute for Global Health and Development, Peking University, Beijing, China; 3 Independent Diagnostics and Health Systems Expert, Geneva, Switzerland

**Keywords:** Diagnostic tools, Utilization, Disparity, COVID-19, Pandemic

## Abstract

**Background:**

Disparities in the utilization of essential medical products are a key factor contributing to inequality in health outcomes. We aimed to analyze the trends and influencing factors in using Coronavirus disease 2019 (COVID-19) diagnostic tools and disparities in countries with different income levels.

**Methods:**

We conducted a cross-sectional study using open and publicly available data sources. Data were mainly collected from the Foundation for Innovative New Diagnostics, "Our World in Data," and the Global Burden of Disease databases. Negative binomial regression model and generalized linear mixed model were employed to investigate into five sets of factors associated with the usage of diagnostics: severity of COVID-19, socioeconomic status, health status, medical service capacity, and rigidity of response. Dominance analysis was utilized to compare the relative importance of these factors. The Blinder–Oaxaca decomposition was used to decompose the difference in the usage of diagnostics between countries.

**Results:**

The total COVID-19 testing rate ranged from 5.13 to 22,386.63 per 1000 people from March 2020 to October 2022 and the monthly testing rate declined dramatically from January 2022 to October 2022 (52.37/1000 vs 5.91/1000).. The total testing rate was primarily associated with socioeconomic status (37.84%), with every 1 standard deviation (SD) increase in Gross Domestic Product per capita and the proportion of people aged ≥ 70, the total testing rate increased by 88% and 31%. And so is the medical service capacity (33.66%), with every 1 SD increase in health workforce density, the number increased by 38%. The monthly testing rate was primarily associated with socioeconomic status (34.72%) and medical service capacity (28.67%), and the severity of COVID-19 (21.09%). The average difference in the total testing rates between high-income and low-income countries was 2726.59 per 1000 people, and 2493.43 (91.45%) of the differences could be explained through the five sets of factors.

**Conclusions:**

Redoubling the efforts, such as local manufacturing, regulatory reliance, and strengthening the community health workforce and laboratory capacity in low- and middle-income countries (LMICs) cannot be more significant for ensuring sustainable and equitable access to diagnostic tools during pandemic.

**Supplementary Information:**

The online version contains supplementary material available at 10.1186/s41256-023-00330-1.

## Introduction

Promoting health equity and reducing disparities is one of the cores Sustainable Development Goals [1]. Health disparities are defined as the differences in health outcomes or healthcare use between distinct vulnerable and less vulnerable populations [2]. During the Coronavirus disease 2019 (COVID-19) pandemic, evidence shows that ensuring adequate and timely access to diagnostics has played a crucial role in maintaining the functioning of the economy, managing healthcare demand, and effectively suppressing transmission [3]. Testing is an essential component of an effective response strategy to the pandemic [4–6].

Ensuring equitable distribution and access to COVID-19 diagnostic tools is essential for transitioning from an acute response to long-term COVID-19 management [7]. More importantly, well-established mechanisms and systems will be instrumental in the global response to the next pandemic. The Access to COVID-19 Tools Accelerator (ACT-A) set a target of 1 test per 1000 people per day in low- and middle-income countries (LMICs) in 2021 [8]. However, the majority of LMICs around the world still do not meet the required COVID-19 test volumes [9]. There are significant disparities in the use of diagnostics tools between countries with different income levels, with the number of tests per 1000 people in high-income countries (HICs) being more than ten times higher than that in LMICs. Multiple blockades impede the wide usage and distribution of diagnostics, including inadequate supply of testing products, high costs, limited testing sites, and insufficient national attention [10, 11].

A timely analysis of the utilization and distribution of diagnostics during the COVID-19 pandemic, along with a thorough examination of the key factors influencing disparities in the usage, is a critical pathway to promoting equitable access to diagnostics and addressing future pandemic [12]. Here are the key questions: when the next pandemic breaks out, will it be possible to promptly develop and scale up the production of reliable, affordable diagnostics to cover the countries in need? Moreover, how can the capacity of LMICs be improved to roll out tests, and reply on epidemiological data to effectively tackle the pandemic?

Previous studies mainly explored disparities in the usage of COVID-19 diagnostics among various racial groups [13, 14], socioeconomic areas [15], and individuals with varying COVID-19 risk profiles [3]. To the best of our knowledge, there has been no study yet to systematically examine the utilization of COVID-19 diagnostics at the global, regional, and national level. The current literature still does not adequately address the uneven distribution and access to COVID-19 diagnostics through a comprehensive analysis focusing on the impact of multi-level factors. Therefore, we aimed to analyze the global, regional, and national trends and influencing factors in the usage of diagnostic tools since the outbreak of COVID-19. We further analyzed disparities in using COVID-19 diagnostic tools at various income levels and to gauge to what extent influencing factors may contribute to these disparities.

## Methods

### Study design and data set

We conducted a cross-sectional study using open and publicly available data sources. Specifically, the data on the COVID-19 diagnostics used, including the polymerase chain reaction (PCR) and antigen tests, were mainly sourced from the Foundation for Innovative New Diagnostics (FIND). Considering the accessibility of data, we analyzed the usage data from March 1, 2020 to October 31, 2022. Countries were categorized into four regions based on their sociodemographic index (SDI) and six WHO regions according to geographic contiguity. SDI is a composite indicator that reflects a country’s socio-demographic level. More information about the distribution of SDI is provided in Additional file 1. Data concerning factors associated with the utilization of COVID-19 diagnostic tools encompassed five sets of country-level indicators, including the severity of COVID-19, socioeconomic status, health status, medical service capacity, and rigidity of response. Evidence of COVID-19's dynamic severity and rigidity of response was primarily collected from “Our World in Data” with recent diagnostics data from different governments. Data on socioeconomic status, health status, and medical service capacity were obtained from the Global Health Data Exchange query tool with relevant data since 2019. Detailed descriptions of data collection methods and data sources are provided in Additional file 2.

This study was conducted from November 1, 2022 to February 28, 2023. We used standardized country names to link the multilevel datasets and excluded countries with missing values on key variables. Ultimately, 161 countries and territories were included in our study. This study used publicly available data and was deemed exempt from guidelines for human research from the Institutional Review Board of Peking University.

### Outcome variables

The testing rate used in our study was determined by calculating the number of tests procured per 1000 people for each country. We defined the total testing rate as the cumulative number of tests procured per 1000 people for each country, which encompassed the total nationwide usage per 1000 people from March 1, 2020, to October 31, 2022, including both PCR and antigen tests. The study period was chosen for several reasons, including the high infection rates of the Omicron variant, variations in the frequency of testing data reporting among many countries, and the different types of tests used, which resulted in varying reporting methods between countries, potentially affecting the accuracy of testing data. In addition, to investigate the changes and time trends in testing rates, we also analyzed the total testing rate for the last twelve months (from November 1, 2021, to October 31, 2022) and the monthly testing rate (cumulative number of COVID-19 tests per 1,000 people every month). The monthly testing rate refers to the cumulative number of tests per 1,000 people for each month.

### Explanatory variables

Five sets of factors associated with the usage of COVID-19 diagnostics were analyzed. The severity of COVID-19 was measured by the number of deaths and cases per 100,000 people, both in total and on a monthly basis according to the outcome variables. Socioeconomic status was assessed by Gross Domestic Product (GDP) per capita and the proportion of people aged ≥ 70. The health status was characterized by the prevalence of cardiovascular diseases, chronic respiratory diseases, diabetes diseases and neoplasms per 100,000 people [16]. The medical service capacity was quantified by using the health workforce density of per 100,000 people. The rigidity of response was measured by using the stringency index, a composite measure encompassing nine response metrics: school closures, workplace closures, cancellation of public events, restrictions on public gatherings, closures of public transport, stay-at-home requirements, public information campaigns, restrictions on internal movements, and international travel controls [17]. To better illustrate the result comparison, we computed standardized Z scores by subtracting the mean of the independent variables from each variable value and then dividing it by the standard deviation (SD).

### Statistical analysis

Descriptive statistical analyses were conducted to describe the usage of COVID-19 diagnostics across the world. A negative binomial regression model was used to investigate into five sets of factors associated with the total usage and a generalized linear mixed model was used to investigate the impact of these same factors on monthly usage, with all influencing factors simultaneously included in the model. To assess the relative importance of the five sets of factors associated with the total testing rate or testing rate per month, we conducted a dominance analysis for decomposition. The dominance statistics were used as an index of effect size [18]. The Blinder–Oaxaca decomposition technique for nonlinear models was employed to decompose the differences in the usage between low-income countries (categorized as low or lower-middle countries by SDI) and high-income countries (categorized as high or upper-middle countries by SDI), thus elucidating the determinants of these disparities [19].

All associations were presented as incidence rate ratio (IRR) or coefficients with corresponding 95% confidence intervals (Cis). A two-sided *p*-value < 0.05 was considered statistically significant. Stata version 16.0 for Mac (Stata Corp, College Station, TX, USA) and R Studio Version 1.2.5042 (The R Project for Statistical Computing, Vienna, Austria) were used for the statistical analyses.

## Results

### Global, regional and national trends of the usage of COVID-19 diagnostics

**Fig. 1:**
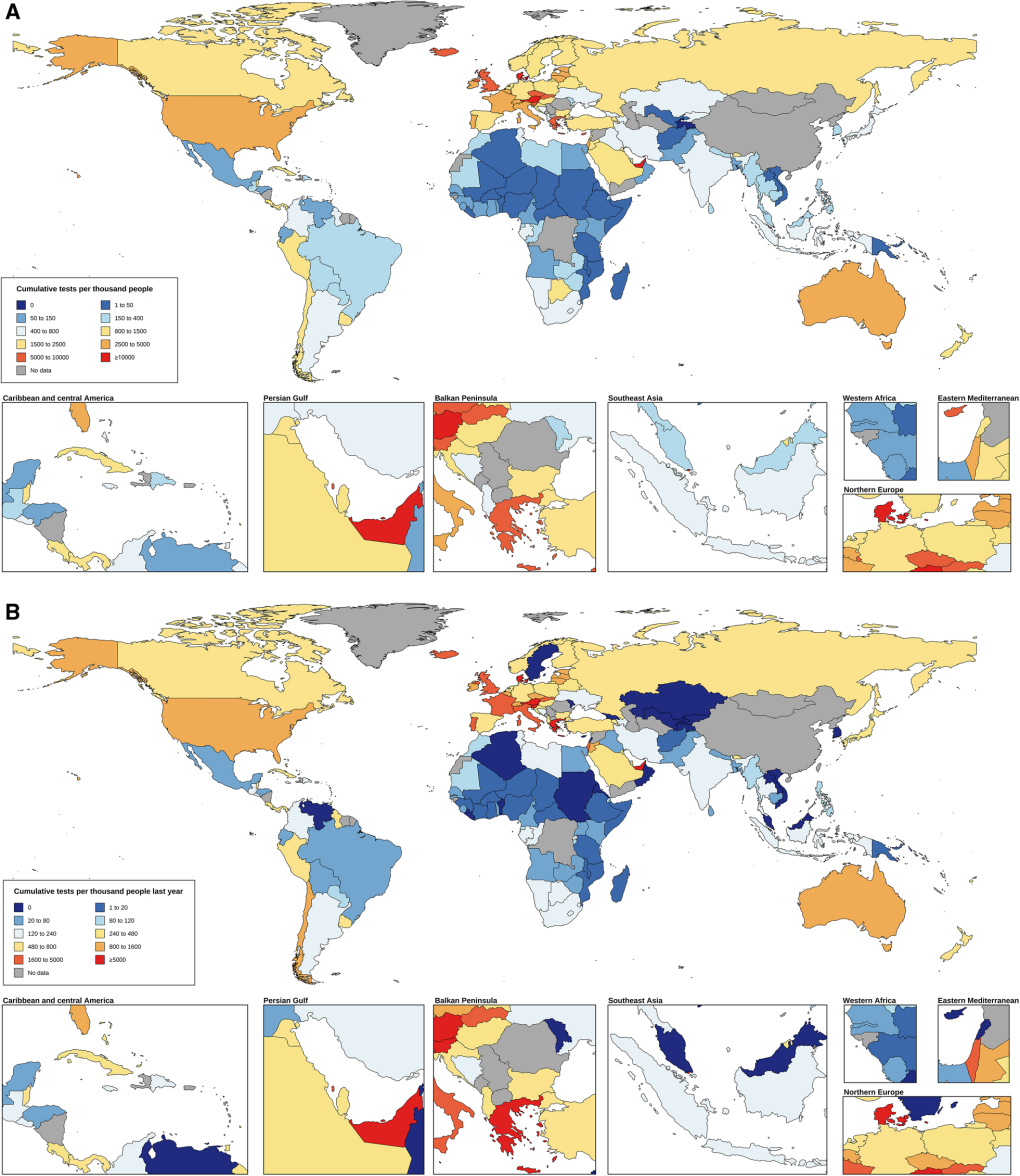
Total testing rate per thousand people across the world. **A** From March 2020 to October 2022. **B** From November 2021 to October 2022

Globally, the total testing rate of COVID-19 was 491.25 per 1000 people from March 1, 2020, to October 31, 2022, while the number was 128.01 per 1000 people from November 1, 2021 to October 31, 2022. The total testing rate of COVID-19 per 1000 people in high SDI regions was 72 times higher than that in low SDI regions. Across the six WHO regions, the European region had the highest total testing rate (2102.25 per 1000 people), whereas the African region had the lowest total testing rate (73.84 per 1000 people). Similar trends were found in the last twelve months **(**Table 1**).** For 161 countries and territories, the total testing rate ranged from 5.13 to 22,386.63 per 1000 people from March 2020 to October 2022 and 3.03 to 11,507.57 per 1000 people in the last twelve months among different countries **(**Fig. 1**).**

**Table 1:** Total tests per 1000 people from 2020 to 2022 in different regions

	Median (q1, q3)	
	2020.03–2022.10	2021.11–2022.10
Overall	491.25 (130.77, 1616.42)	128.01 (19.57, 660.81)
SDI region		
Low	35.28 (17.34, 62.57)	11.59 (4.27, 24.45)
Lower middle	173.70 (73.8, 400,9)	52.21 (19.57, 109.79)
Upper middle	706.70 (384.44, 1118.49)	178.73 (78.70, 421.10)
High	2540.79 (1317.22, 5344.70)	885.91 (412.70, 1672.75)
WHO region		
African	73.84 (35.28, 212.35)	23.42(9.03, 109.38)
Americas	626.19 (343.34, 1436.33)	237.22 (90.76, 587.61)
Eastern Mediterranean	401.55 (112.15, 1499.29)	104.26 (10.56, 448.48)
European	2102.25 (942.17, 4275.31)	749.51 (205.13, 1594.20)
South-East Asian	357.64 (195.00, 635.75)	83.88 (75.71, 206.50)
Western pacific	305.00 (63.12, 1466.28)	58.66 (0.00, 421.74)

**Fig. 2:**
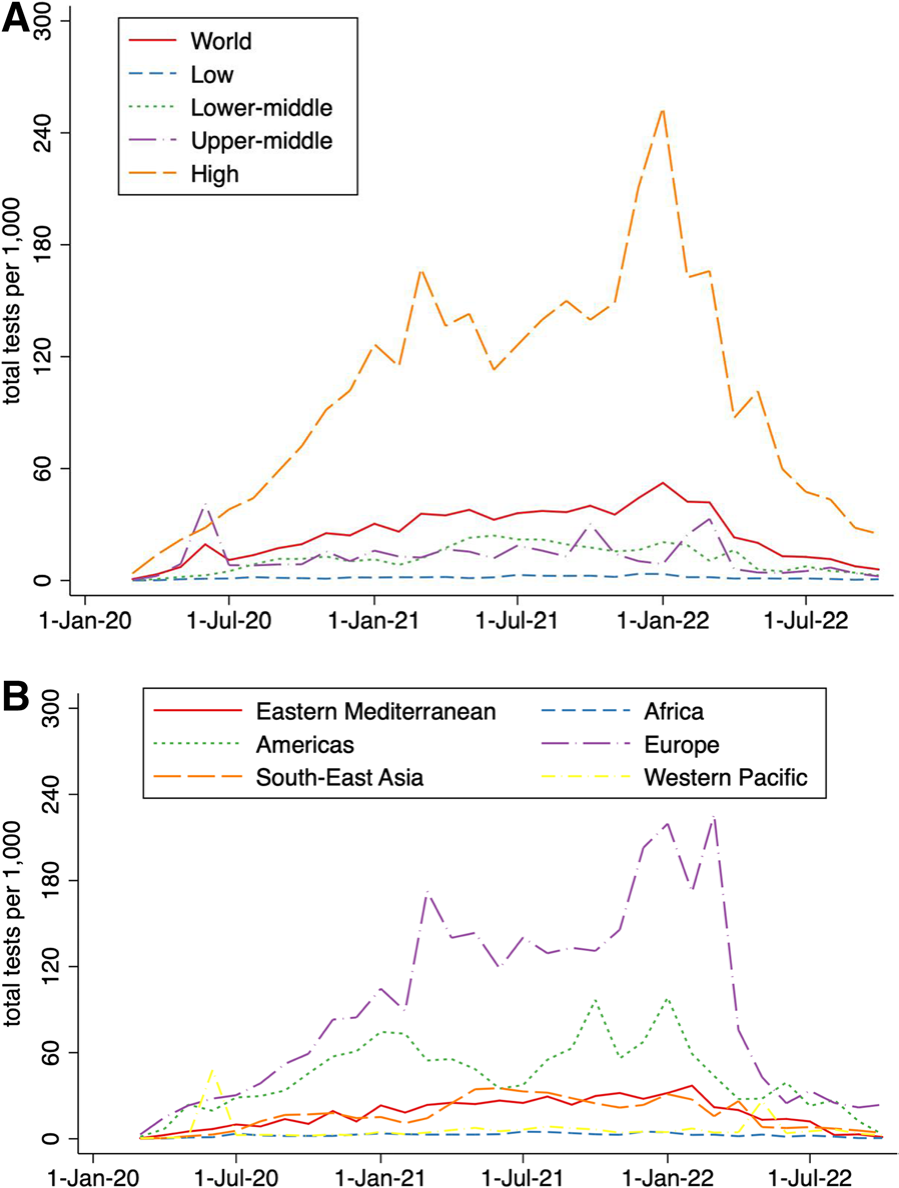
Monthly testing rate across different regions. **A** Monthly testing rate by different income level regions from March 2020 to October 2022. **B** Monthly testing rate by different WHO regions from March 2020 to October 2022

The regional monthly testing rate showed a fluctuating trend with peaks around March 2021 and January 2022 for high-income countries (167.31/1000 and 253.70/1000). The peak for monthly testing at the global level was in January 2022 with the number of 52.37 per 1000 people. These peaks occurred when the Delta and Omicron variants swept the world, respectively. However, from January 2022, the monthly testing rate in each country declined dramatically in recent months with the spread of Omicron from January 2022 to October 2022 (52.37/1000 vs 5.91/1000) **(**Fig. 2**).**

### Factors associated with total testing rate and monthly testing rate

The total testing rate was primarily associated with the socioeconomic status (37.84%), medical service capacity (33.66%), health status (20.84%), and severity of COVID-19 (7.31%). For every 1 SD increase in GDP per capita and proportion of the people aged ≥ 70, the number of total tests increased by 88% and 31% (IRR:1.88, 95% CI 1.78–1.98; IRR: 1.31, 95% CI 1.22–1.40). The total testing rate increased by 1.37 (IRR: 1.38, 95% CI 1.30–1.47) with every 1 SD increase in health workforce density. Additionally, for every 1 SD increase in the prevalence of cardiovascular diseases, chronic respiratory diseases and neoplasms, the number of total testing rate increased by 21% (IRR: 1.21, 95% CI 1.13–1.30), 10% (IRR: 1.10, 95% CI 1.07–1.13) and 13% (IRR: 1.13, 95% CI 1.10–1.16), respectively. The monthly testing rate was primarily associated with socioeconomic status (34.72%) and medical service capacity (28.67%), and the severity of COVID-19 (21.09%) **(**Table 2**).**

**Table 2:** Factors associated with total testing rate and monthly testing rate

Factors	Total testing rate			Monthly testing rate		
	Standard dominance statistic	IRR	95% CI	Standard dominance statistic	Coefficients	95% CI
Severity of COVID-19						
Total/monthly incident rate	7.31	1.34	(1.29 to 1.40)	21.09	32.87	(26.55 to 39.20)
Total/monthly mortality rate		1.07	(1.03 to 1.11)		7.58	(1.05 to 14.10)
Socioeconomic status						
GDP per capita	37.84	1.88	(1.78 to 1.98)	34.72	24.99	(4.19 to 45.80)
Proportion of age ≥ 70		1.31	(1.22 to 1.40)		10.22	(4.41 to 16.03)
Health status						
Cardiovascular diseases	20.84	1.21	(1.13 to 1.30)	16.44	−8.09	(−41.99 to 25.81)
Diabetes diseases		0.85	(0.83 to 0.88)		−2.80	(−16.22 to 10.62)
Chronic respiratory diseases		1.10	(1.07 to 1.13)		10.98	(−2.36 to 24.33)
Neoplasms		1.13	(1.10 to 1.16)		7.37	(−5.62 to 20.36)
Medical service capacity						
Health workforce density	33.66	1.38	(1.30 to 1.47)	28.67	24.29	(−3.23 to 51.81)
Rigidity of response						
Stringency index	0.35	1.25	(1.21 to 1.28)	−0.92	10.22	(4.41 to 16.03)

### Blinder–Oaxaca decomposition of difference in total testing rate

The average difference in total testing rate between high-income and low-income countries was 2726.59 per 1000 people, and 2493.43 per 1000 people (91.45%) of the differences could be explained through the differences of five sets of factors. The main indicators that explained the differences were GDP per capita (31.66%), the proportion of people aged ≥ 70 (30.37%), the health workforce density (24.58%), and the total incidence rate (9.95%) **(**Table 3**).**

**Table 3:** The Blinder–Oaxaca decompose of total testing rate difference between low-income and high-income countries

Factors	Difference in characteristics		Percentage (%)	Difference in coefficients		Percentage (%)
	Coefficients	95%CI		Coefficients	95%CI	
Severity of COVID-19						
Total incident rate	271.22	(224.35 to 318.09)	9.95	169.66	(129.96 to 209.36)	6.22
Total mortality rate	−76.77	(−125.79 to −27.75)	−2.82	−18.98	(−41.13 to 3.17)	−0.70
Socioeconomic status						
GDP per capita	863.25	(745.01 to 981.49)	31.66	250.81	(159.19 to 342.44)	9.20
Proportion of age > 70	828.09	(669.10 to 987.09)	30.37	−95.10	(−140.24 to −49.96)	−3.49
Health status						
Cardiovascular diseases	−83.98	(−230.00 to 62.04)	−3.08	46.58	(14.66 to 78.50)	1.71
Chronic respiratory diseases	25.96	(14.05 to 37.88)	0.95	0.38	(−1.05 to 1.80)	0.01
Diabetes diseases	44.96	(36.50 to 53.42)	1.65	−1.36	(−2.63 to −0.09)	−0.05
Neoplasms	−53.13	(−65.57 to −40.69)	−1.95	3.04	(1.54 to 4.53)	0.11
Medical service capacity						
Health workforce density	670.32	(538.49 to 802.14)	24.58	126.11	(67.86 to 184.37)	4.63
Rigidity of response						
Stringency index	3.51	(2.30 to 4.73)	0.13	−0.03	(−0.05 to −0.02)	−0.00
Total differences	2726.59					
Explained (due to characteristics)	2493.43 (91.45%)					
Unexplained (due to coefficients)	233.15 (8.55%)					

## Discussion

COVID-19 testing is the foundation of treatment, and timely testing can effectively reduce morbidity and mortality. Enhancing access to testing will improve surveillance, monitor emerging variants, and guide strategies to end the pandemic [20]. As far as we know, this is an exploratory study to analyze differences in the usage and identify the factors associated with the differences at the global, regional and national level. We found significant disparities in the usage in different countries, with a notable decline in testing worldwide in recent months. Our findings highlight that multiple strategies are urgently needed to mitigate the disparities in distribution and access to COVID-19 diagnostics.

We found that both total testing rate and monthly testing rate were associated with the socioeconomic factors. According to WHO, most manufacturers granted Emergency Use Listing (EUL) status for COVID-19 diagnostic tools are located in middle- and high-income countries [21] (Additional file 3). Previous studies have also confirmed that COVID-19 testing rates are significantly higher in high-income populations than in low-income ones [4]. Additionally, the proportion of the elderly population significantly contributed to the overall testing volume. This is likely due to the fact that COVID-19 is a greater health threat to the elderly [22], and the middle-aged and elderly are generally more concerned about COVID-19 [23].

The health workforce played a critical role in the utilization of COVID-19 response tools. Whether it was a diagnostic reagent or a product such as a vaccine, a shortage of health workforce could undermine the efforts to reach populations in dire need of these resources [24]. The COVID-19 pandemic further highlights the global health workforce shortage and the need to strengthen the health workforce in LMICs as a priority issue in global health today [25, 26]. In addition, we found that health status, especially the prevalence of respiratory diseases and cancer, was significantly and positively correlated with the volume of testing. This may be related to the higher risk of developing serious illness or death after COVID-19 infection in patients with respiratory diseases and cancer [27, 28]. Our results indicated that the severity of COVID-19, had a significant effect on both total and monthly testing rate. Compared with the total testing rate, the proportion explained by severity of COVID-19 was higher in monthly testing rate. The results were consistent with the previous study, which indicated that high-risk settings need more routine viral testing to prevent outbreaks and reduce the incidence of COVID-19 [29].

There is a large difference in overall COVID-19 testing between high-income and low-income countries, with more than 90% of the difference due to differences in GDP per capita, the proportion of the elderly population, the health workforce density, and the number of COVID-19 cases. Reducing the differences in economic development and health workforce numbers can effectively improve the equity of COVID-19 testing [30]. Although the number of prevalent cases is higher in high-income countries, there is a need to be alert to the possibility that the difference in the number of prevalent cases between high- and low-income countries is underestimated. A large number of cases went undetected in developing countries due to insufficient testing.

For the current analysis of the disparities of COVID-19 diagnostic reagents, it is more important to propose practical approaches to responding to the next pandemic. We call for treating diagnostic reagents as a global public good to meet all needs, not just a few [31]. Addressing the inequity of diagnostic reagents requires a systematic thinking in four dimensions: research and development, production, regulation, and delivery. For research and development, stakeholders, such as WHO, FIND, UNICEF and countries concerned need to establish an effective collaboration mechanism to advance the development of diagnostic reagents, build an emergency and normalized response strategy, and ensure the large-scale supply of quality-assured diagnostics through market-shaping interventions. For production, technology transfer to LMICs and capacity building are needed to facilitate the local production of diagnostic tools through the patent pool, product development partnerships and other possible means. International organizations, such as WHO and FIND, may organize experts to provide procedural guidance to companies ready to be involved. For regulation, technical guidance on the WHO EUL and prequalification (PQ) given by professionals and promoting regulatory reliance on diagnostic reagents in areas lacking regulatory capacity are desired [32]. For delivery, the future direction of health assistance should focus on strengthening the construction of laboratory systems, training health personnel and developing modern supply chain systems in recipient countries.

Our study results still have several limitations. First, there are different decomposition methodologies, so that the results may differ depending on the method used. Second, this was an ecological study that utilized aggregate country-level data. There exist more individual-level factors that may influence COVID-19 testing not taken into account here. Third, the accessibility of testing was also a key factor affecting the usage of testing at the beginning of the pandemic. However, due to data limitations, we could not include it in this study. Fourth, the observational nature of this study limited our ability to draw any causal inference from the findings. Fifth, the total and monthly testing rate used in our study was a rough surrogate for testing, as it was based on the number of procured per 1000 people for each country. Sixth, the data used in our study were from the country level, and we could not analyze trends and variations in the use of diagnostic reagents within a specific country. Seventh, we could not distinguish the number of tests between what is used for screening and what is used for diagnosis. Future studies with more specific data about diagnostic tools for screening and diagnosis are needed to further investigate disparities in using COVID-19 diagnostics at various income levels.

## Conclusions

Different countries exhibited significant disparities in the usage of COVID-19 diagnostics, often attributable to underlying factors. Testing is a fundamental component of test-to-treat strategies and serves as the first line of defense in response to future pandemics. Ensuring cooperation and alignment among relative stakeholders is the key to incentivizing and sustaining the development, supply and deployment of quality-assured and affordable diagnostics. Intensifying efforts such as local manufacturing, regulatory reliance, and strengthening the community health workforce and laboratory capacity in LMICs cannot be overemphasized in guaranteeing sustainable and equitable access to diagnostic tools in the event of future pandemics.

### Supplementary Information

**Additional file 1**. The list of countries and territories by SDI level.

**Additional file 2**. Data collection methods and data sources.

**Additional file 3**. The distribution of COVID-19 IVD Manufacturing country granted WHO EUL status from 1st March 2020 - 31th October 2022.

## Data Availability

Data in this study are available in a public, open access repository.

## Abbreviations

COVID-19: Coronavirus disease 2019
WHO: World Health Organization
UNICEF: United Nations Children’s Fund
FIND: Foundation for Innovative New Diagnostics
ACT-A: Access to COVID-19 Tools Accelerator
GBD: Global Burden of Disease
SD: Standard deviation
GDP: Gross Domestic Product
LMICs: Low- and middle-income countries
HICs: High-income countries
PCR: Polymerase chain reaction
SDI: Sociodemographic index
EUL: Emergency using list
PQ: Prequalification
