# Supplementary material for: Factors associated with the utilization of diagnostic tools among countries with different income levels during the COVID-19 pandemic
Source: Glob Health Res Policy. 2023 Oct 27;8:45. doi: 10.1186/s41256-023-00330-1 (PMC10605783; doi:10.1186/s41256-023-00330-1)
Supplement: Supplementary file 1 — Additional file 1. The list of countries and territories by SDI level. [file 41256_2023_330_MOESM1_ESM.docx]

**Additional file 1: The list of countries and territories by SDI level**

| SDI | Countries and territories |
| --- | --- |
| Low | Afghanistan, Burkina Faso, Burundi, Central African Republic, Chad, Congo, Eritrea, Ethiopia, Gambia, Guinea, Liberia, Madagascar, Malawi, Mali, Mozambique, Niger, Rwanda, Sierra Leone, Somalia, South Sudan, Sudan, Togo, Uganda |
| Lower-middle | Algeria, Angola, Bangladesh, Belize, Benin, Bhutan, Bolivia, Cambodia, Cameroon, Cape Verde, Comoros, Cote d'Ivoire, Djibouti, Egypt, El Salvador, Eswatini, Ghana, Honduras, India, Indonesia, Iran, Kenya, Kyrgyzstan, Laos, Lesotho, Mauritania, Morocco, Myanmar, Nepal, Nigeria, Pakistan, Palestine, Papua New Guinea, Philippines, Senegal, Sri Lanka,  Tajikistan, Tanzania, Tunisia, Ukraine, Uzbekistan, Venezuela, Vietnam, Zambia, Zimbabwe |
| Upper-middle | Albania, Argentina, Armenia, Azerbaijan, Belarus, Bosnia and Herzegov, Botswana, Brazil, Bulgaria, China, Colombia, Costa Rica, Cuba, Dominican Republic, Ecuador, Equatorial Guinea, Fiji, Gabon, Georgia, Grenada, Guatemala, Guyana, Iraq, Jamaica  Jordan, Kazakhstan, Lebanon, Libya, Malaysia, Maldives, Mauritius, Mexico, Moldova, Namibia, Panama, Paraguay, Peru, Romania, Russia, South Africa, Thailand, Turkey |
| High | Antigua and Barbuda, Australia, Austria, Bahamas, Bahrain, Barbados, Belgium, Brunei, Canada, Chile, Croatia, Cyprus, Czechia, Denmark, Estonia, Finland, France, Germany, Greece, Hungary, Iceland, Ireland, Israel, Italy, Japan, Kuwait, Latvia, Lithuania, Luxembourg, Malta, Netherlands, New Zealand, Norway, Omanl, Poland, Portugal, Qatar, Saudi Arabia, Seychelles, Singapore, Slovakia, Slovenia, South Korea, Spain, Sweden,  Switzerland, Taiwan, Trinidad and Tobago, United Arab Emirates, United Kingdom, United States, Uruguay |
