# Supplementary material for: Factors associated with the utilization of diagnostic tools among countries with different income levels during the COVID-19 pandemic
Source: Glob Health Res Policy. 2023 Oct 27;8:45. doi: 10.1186/s41256-023-00330-1 (PMC10605783; doi:10.1186/s41256-023-00330-1)
Supplement: Supplementary file 2 — Additional file 2. Data collection methods and data sources. [file 41256_2023_330_MOESM2_ESM.docx]

**Additional file 2: Data collection methods and data sources**

*Data merged process*

We used standardized country names to link the diagnostic usage data from the Foundation for Innovative New Diagnostics (FIND), "Our World in Data," and the GBD database. First, we standardized the country names from each database. Then, we merged the databases using the country names as the key variable. Finally, we excluded countries with missing values on important variables. Ultimately, 161 countries and territories were included in our study.

*Outcome metrics included in our study*

The data for outcome metrics were from the Foundation for Innovative New Diagnostics (FIND).

1. **Total testing rate**. The cumulative administered number of COVID-19 tests per 1,000 people from March 1, 2020 to October 31, 2022, which included the polymerase chain reaction (PCR) and antigen tests.
2. **Total testing rate in last year**. The cumulative administered number of COVID-19 tests per 1,000 people from November 1, 2021 to October 31, 2022, which included the polymerase chain reaction (PCR) and antigen tests.
3. **Monthly testing rate**. The cumulative administered number of COVID-19 tests per 1,000 people every month, which included the polymerase chain reaction (PCR) and antigen tests.

**Table S1. The list of variables for investigating associations with total/ monthly testing rate**

| **Variables** | **Units** | **Temporal coverage** | **Source** |
| --- | --- | --- | --- |
| Severity of COVID-19 |  |  |  |
| Total/Monthly cases rate | Number of tests per 1,000 | March 1, 2020- October 31, 2022 | Our World in Data |
| Total/Monthly deaths rate | Number of tests per 1,000 | March 1, 2020- October 31, 2022 | Our World in Data |
| Socioeconomic status |  |  |  |
| GDP per capita | Purchasing power parity-adjusted dollars | 2019 | Our World in Data |
| Proportion of age≥70 | Percentage of people aged more than 70 | 2019 | Our World in Data |
| Health status |  |  |  |
| Cardiovascular diseases | Number of patients per 100,000 | 2019 | GBD 2020 |
| Diabetes diseases | Number of patients per 100,000 | 2019 | GBD 2020 |
| Chronic respiratory diseases | Number of patients per 100,000 | 2019 | GBD 2020 |
| Neoplasms | Number of patients per 100,000 | 2019 | GBD 2020 |
| Medical service capacity |  |  |  |
| Health workforce density | Number of health workforce per 10,000 | 2019 | GBD 2020 |
| Rigidity of response |  |  |  |
| Stringency index | Index | March 1, 2020- October 31, 2022 | Our World in Data |
