# Supplementary material for: Factors associated with the utilization of diagnostic tools among countries with different income levels during the COVID-19 pandemic
Source: Glob Health Res Policy. 2023 Oct 27;8:45. doi: 10.1186/s41256-023-00330-1 (PMC10605783; doi:10.1186/s41256-023-00330-1)
Supplement: Supplementary file 3 — Additional file 3. The distribution of COVID-19 IVD Manufacturing country granted WHO EUL status from 1st March 2020 - 31th October 2022. [file 41256_2023_330_MOESM3_ESM.docx]

**Additional file 3 The distribution of COVID-19 IVD Manufacturing country granted WHO EUL status from 1^st^ March 2020 - 31^th^ October 2022**

**
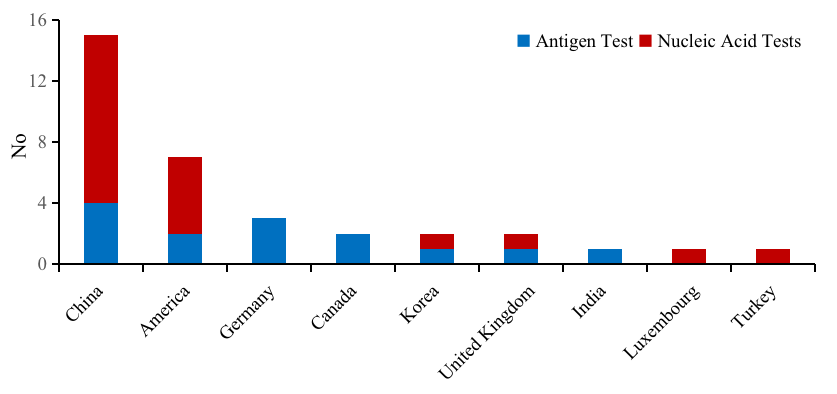
**
